# A method for assessing implementation success of a peer-led suicide prevention program

**DOI:** 10.1186/1748-5908-10-S1-A42

**Published:** 2015-08-20

**Authors:** Peter A Wyman, Mariya Petrova, Karen Schmeelk-Cone, Nathaniel Kerr, Anthony Pisani, C Hendricks Brown, Lisa Saldana, Trevor Pickering, Thomas Valente

**Affiliations:** 1 School & Community-Based Prevention Program, Department of Psychiatry, University of Rochester School of Medicine, Rochester, NY 14642, USA; 2 Center for Prevention Implementation Methodology (Ce-PIM), Northwestern University, Feinberg School of Medicine, Chicago, IL 60611, USA; 3 Oregon Social Learning Center, Eugene, OR 97401, USA; 4 Institute for Prevention Research, Department of Preventive Medicine, Keck School of Medicine, USC, Los Angeles, CA 90034, USA

## Objective

To summarize a first stage of research on implementation of a peer leader suicide prevention program by testing the utility of a method for tracking and reporting each school's success in retaining and preparing Peer Leaders.

## Background

Peer leader programs that prepare opinion leaders to spread healthy practices through their social networks reduce high-risk sex behaviors and show promise in preventing adolescent substance use and suicidal behavior. However, knowledge of implementation processes is very limited. To address this limitation, we drew on the Stages of Implementation Completion (SIC) framework to measure a key phase of peer leader implementation.

## Methods

40 high schools were randomly assigned to either immediate Sources of Strength (n = 20) or waitlist (n = 20). The schools were underserved by mental health services and over-represented by youth at high risk for suicide (e.g., American Indians). In the 20 implementing schools, 656 students (18-71 per school) received Peer Leader (PL) training. Adult mentors facilitated PL meetings to reinforce program concepts and help PLs plan and execute activities to spread healthy coping practices. Using a framework derived from the Stages of Implementation Completion (SIC), school reports of PL meeting dates/attendance were codified as indices of school success in retaining and preparing PLs. Surveys with 5,712 students showed wide school-level variation in success of PLs in reaching their classmates with the prevention concepts. In analytic models examining predictors of school-level exposure, a higher proportion of student population trained as PLs and greater retention of PLs predicted higher population exposure to the prevention program, congruent with diffusion of innovations theory, whereas frequency of meetings did not.

## Contribution to the field

Identified an efficient method (derived from the SIC) for assessing a school's success in preparing/retaining peer leaders. This approach shows promise in providing schools actionable data to increase impact of peer-led programs.

## Funding

NIH R01MH091452.

